# Supplementary material for: Accumulation of STR-Loci Aberrations in Subclones of Jurkat Cell Line as a Model of Tumor Clonal Evolution
Source: Genes (Basel). 2023 Feb 24;14(3):571. doi: 10.3390/genes14030571 (PMC10048572; doi:10.3390/genes14030571)
Supplement: Supplementary file 1 [file genes-14-00571-s001.zip › Table S4. The distribution of Jurkat-B4 subclones between STR profiles.pdf]

Table S4. The distribution of Jurkat-B4 subclones between STR profiles according to growth rate (rapid, medium, slow).

STR-profile	rapid growing clones, n	medium growing clones, n	slow growing clones, n	sum	percentage
N	42	38	28	108	60,0%
12p EM	0	0	11	11	6,1%
12p LOH	0	2	7	9	5,0%
6q EM	6	2	1	9	5,0%
vWA EM	0	4	1	5	2,8%
16q EM	2	1	0	3	1,7%
22q EM	3	0	0	3	1,7%
10q EM	0	1	1	2	1,1%
5q EM	0	1	1	2	1,1%
12+6 EM	0	0	2	2	1,1%
8q LOH	0	1	1	2	1,1%
1,2,12,21,Y LOH	0	2	0	2	1,1%
10q LOH 12p EM	0	0	2	2	1,1%
1q Y LOH	2	0	0	2	1,1%
21q EM	1	0	0	1	0,6%
FGA EM	1	0	0	1	0,6%
10+6 EM	1	0	0	1	0,6%
2+6 EM	1	0	0	1	0,6%
5+5 EM	0	1	0	1	0,6%
7+18 EM	0	1	0	1	0,6%
12+4 EM	0	1	0	1	0,6%
12+22 EM	0	0	1	1	0,6%
12+4+6 EM	0	0	1	1	0,6%
2p LOH	0	1	0	1	0,6%
10q LOH	0	1	0	1	0,6%
22q LOH	1	0	0	1	0,6%
5q LOH	0	1	0	1	0,6%
22q LOH 12p EM	0	0	1	1	0,6%
4q LOH 12pEM	0	0	1	1	0,6%
4q LOH vWA EM	0	0	1	1	0,6%
2p LOH 5q6q EM	0	1	0	1	0,6%
10q5q LOH	0	1	0	1	0,6%
