# Supplementary material for: Accumulation of STR-Loci Aberrations in Subclones of Jurkat Cell Line as a Model of Tumor Clonal Evolution
Source: Genes (Basel). 2023 Feb 24;14(3):571. doi: 10.3390/genes14030571 (PMC10048572; doi:10.3390/genes14030571)
Supplement: Supplementary file 1 [file genes-14-00571-s001.zip › Table S5. The distribution of Jurkat-O1 subclones between STR profiles.pdf]

Table S5. The distribution of Jurkat-O1 subclones between STR profiles according to growth rate (rapid, medium, slow).

STR-profile	rapid growing clones, n	medium growing clones, n	slow growing clones, n	sum	percentage
N	86	83	76	245	64,3%
1q EM	3	5	5	13	3,4%
CSFPO 5q LOH	3	5	3	11	2,9%
8q EM	3	3	3	9	2,4%
10q EM	3	3	3	9	2,4%
6q EM	2	3	3	8	2,1%
22q EM	7	1	0	8	2,1%
vWA EM	2	1	3	6	1,6%
FGA EM	2	1	3	6	1,6%
12p EM	2	4	0	6	1,6%
10q LOH	2	0	4	6	1,6%
21q EM	3	2	0	5	1,3%
16q EM	0	3	1	4	1,0%
7q EM	0	2	1	3	0,8%
2 7 16 TPOX vWA EM	0	0	3	3	0,8%
1q LOH	1	0	2	3	0,8%
1q LOH 6q EM	1	1	1	3	0,8%
CSF 5q EM	2	0	0	2	0,5%
4q LOH	0	1	1	2	0,5%
22q LOH	0	0	2	2	0,5%
18q EM	1	1	0	2	0,5%
16 11 2 Y EM	0	0	1	1	0,3%
TPOH EM	0	1	0	1	0,3%
CSF 5q LOH 8q EM	0	1	0	1	0,3%
CSF 5q LOH 22q EM	0	0	1	1	0,3%
8q vWA EM	0	0	1	1	0,3%
8 16 18 22 EM	1	0	0	1	0,3%
7q LOH 6q EM	0	0	1	1	0,3%
2p LOH	0	0	1	1	0,3%
2p EM	0	0	1	1	0,3%
21q vWA EM	0	1	0	1	0,3%
21q 6q EM	0	1	0	1	0,3%

**2 7 11 TPOX vWA Y EM**	**0**	**0**	**1**	**1**	**0,3%**
**1q LOH CSF 5q 6q EM**	**0**	**0**	**1**	**1**	**0,3%**
**1q LOH 6q EM**	**0**	**0**	**1**	**1**	**0,3%**
**1q 21q EM**	**1**	**0**	**0**	**1**	**0,3%**
**13q TPOX EM**	**0**	**0**	**1**	**1**	**0,3%**
**13q 18q EM**	**0**	**1**	**0**	**1**	**0,3%**
**13q 16q EM**	**0**	**1**	**0**	**1**	**0,3%**
**12p 6q EM**	**1**	**0**	**0**	**1**	**0,3%**
**12p 4q EM**	**0**	**0**	**1**	**1**	**0,3%**
**11p Y EM**	**0**	**0**	**1**	**1**	**0,3%**
**10q vWA EM**	**1**	**0**	**0**	**1**	**0,3%**
**10q LOH 8q EM**	**0**	**1**	**0**	**1**	**0,3%**
**10q LOH 21q EM**	**0**	**0**	**1**	**1**	**0,3%**
**10q LOH 12p EM**	**0**	**1**	**0**	**1**	**0,3%**
